# Interactions of Ag Particles Stabilized by 7,7,8,8-Tetracyanoquinodimethane with Olefin Molecules in Poly(ether-block-amide)

**DOI:** 10.3390/molecules27134122

**Published:** 2022-06-27

**Authors:** Minsu Kim, Younghyun Cho, Sang Wook Kang

**Affiliations:** 1 Department of Chemistry, Sangmyung University, Seoul 03016, Korea; ms19942002@gmail.com; 2 Department of Energy Systems Engineering, Soonchunhyang University, Asan 31538, Korea; 3 Department of Chemistry and Energy Engineering, Sangmyung University, Seoul 03016, Korea

**Keywords:** silver nanoparticles, electron acceptors, poly(ether-block-amide) (PEBAX), 7,7,8,8-tetracyanoquinodimethane (TCNQ), olefin separation

## Abstract

In this study, to use a stabilized carrier, silver nanoparticles (AgNPs) were used as carriers and electron acceptors were added to activate the surface of AgNPs as olefin carriers. In addition, poly(ether-block-amide) (PEBAX), consisting of polyamide (hard segments) and polyether (soft segments), was investigated for the correlation of the between-segments ratio related to the stability of AgNPs and separation performance. As a result, contrary to the expectation that high permeance would be observed in PEBAX-1657/AgNPs/7,7,8,8-tetracyanoquinodimethane (TCNQ) membrane, which had a higher ratio of polyether soft segment, the PEBAX-5513/AgNPs/TCNQ membrane, which had a relatively high proportion of polyamide, showed a higher permeance without difference in selectivity. These unexpected data were attributable to the fact that the relatively abundant amount of PA groups in PEBAX-5513 was able to stabilize and positively polarize the surface of AgNPs, resulting in the stabilized and high performance of olefin separation.

## 1. Introduction

As global demand for pure olefins increases, there has been a great advantage in looking for a reliable alternative to separating olefins from paraffins that replaces conventional energy-consuming and costly distillation methods [1]. As mentioned above, the conventional distillation method has consumed a great deal of cost and energy in the separation process, and the cost of constructing the distillation column is also costly and complicated, so it is urgent to develop an alternative separation method. Therefore, new inorganic chemical techniques such as zeolitic imidazolate framework (ZIF) and metal organic framework (MOF) have been recently researched to replace inefficient processes [2,3,4,5,6,7]. Furthermore, facilitated transfer membranes have recently been spotlighted, owing to the features of increasing selectivity and permeance simultaneously. Among them, separation membranes using silver salts such as AgBF_4_ and AgClO_4_ have been attracting much attention for showing high membrane separation performance even in a solid state [8,9,10]. However, these silver salts tend to be reduced to silver nanoparticles (AgNPs) when added to polyvinylpyrrolidone (PVP) or poly(ethylene oxide) (PEO) [11,12]. These phenomena could increase the obstacles to separation of the polymer electrolyte membrane in long-term operation. In order to overcome the limitations of ion-based polymer membranes, methods of transport using polarized AgNPs capable of reversibly reacting with olefin have been developed [13]. Reversible complexes can be formed by the carrier with a particular component and therefore promote its transport. It not only improves both the selectivity and permeance, but also exhibits stable long-term performance. For example, the composite membranes of poly(ethylene-co-propylene)(EPR)/AgNPs/p-benzoquinone(*p*-BQ) showed separation performance (selectivity: 11 and mixed-gas permeance: 0.5 gas permeation unit (GPU), 1 GPU = 1 × 10^−6^ cm^3^ (STP)/(cm^2^·s·cm Hg)) [14]. For EPR/AgNPs/*p*-BQ composite for olefin separation, the surface-modified AgNPs by *p*-BQ as an electron acceptor could generate high separation performance by facilitated olefin transport.

In a previous study, our group used a PVP/AgNPs nanocomposite membrane using a dipole interface of the AgNPs surface with 7,7,8,8-tetracyanoquinodimethane (TCNQ) [15] The membrane showed unexampled separation performance, selectivity of 50, and permeance of 3.5 GPU [15]. However, higher permeance was required for practical applications, and additional research was conducted using a PEBAX-1657 polymer. Poly(ether-block-amide) PEBAX resin consists of linear chains, which are thermoplastic elastomers [16]. PEBAX consists of two groups: hard and soft groups. The hard polyamide groups could offer a stabilized state for silver nanoparticles by polyamide chains, which is known as an effective stabilizer for nanoparticles, and the flexible polyether groups were known to be offering high permeance by high-chain mobility of the ether linkage [17]. As a result, the mixed-gas permeance largely increased to 10.2 GPU [18,19].

Unfortunately, the relationship by performance varying PA/PE ratios of PEBAX groups has not been yet clear [20,21]. In this study, research was conducted using PEBAX-5513, which has a relatively larger proportion of PA groups (known to be more than 60%) compared with PEBAX-1657 (PE60% and PA40%) to understand the effect of segmenting PE and PA groups in the poly(ether-block-amide) resin. The size and dispersion of AgNPs were characterized by UV-vis, and the coordination interaction of the polymer silver particles was confirmed by IR spectroscopy.

## 2. Materials and Methods

### 2.1. Materials

Polyether block amide-1657 (PEBAX-1657) and polyether block amide-5513 (PEBAX-5513) were supplied from Arkema Inc. (Paris, France), and silver tetrafluoroborate (AgBF_4_, 98%) was purchased from TCI Fine Chemicals (Tokyo, Japan). All chemicals were used as received.

### 2.2. Methods

#### 2.2.1. Preparation of Membranes

PEBAX-1657/AgNPs/TCNQ and PEBAX-5513/AgNPs/TCNQ complex membrane was prepared by 3 wt% of PEBAX-1657 and PEBAX-5513 solution. Each PEBAX was dissolved in a co-solvent that consisted of 7:3 weight ratio of ethanol:water. AgNPs activated by TCNQ were contained in the aforementioned solution. The AgNPs were prepared at a fixed-weight ratio of 1/8.3 to PEBAX-1657 and to PEBAX-5513. The solution was stirred at 75 °C until the solution color became pale yellow in color. Then, the membrane solution was coated onto porous polymer support (polysulfone, Toray Chemical Inc., Seoul, Korea) with RK Control Coater (RK Print-Coat Instruments Ltd., Hertfordshire, UK).

#### 2.2.2. Gas Separation Experiments

Both PEBAX-1657/AgNPs/TCNQ and PEBAX-5513/AgNPs/TCNQ composite membranes were dried in an oven for 1 day at room temperature. Their selectivity for propylene/propane mixture (50/50 vol% of propylene/propane) was confirmed through gas chromatography (GC, Young Lin 6500 model). The rates of the propylene/propane mixture gas flow were controlled by mass flow controllers (MFC). Mixed-gas permeance was measured by bubble flow meter at upstream with various pressure (psig) and atmospheric downstream pressure. Mixed-gas permeance was verbalized as gas permeation unit (GPU), and 1 GPU was 1 × 10^−6^ cm^3^ (STP)/(cm^2^·s·cm Hg).

#### 2.2.3. Characterization

The selective layer of PEBAX-5513/AgNPs/TCNQ composite coated onto polysulfone support was measured by scanning electron microscopy (SEM) purchased from JEOL. IR at resolution of 4 cm^−1^ were measured with FT-IR spectrometer (VERTEX 70 model). UV-vis absorption spectra were investigated through UV-Vis-NIR spectrophotometer (Agilent Technologies Cary 5000) for both PEBAX-1657/AgNPs/TCNQ and PEBAX-5513/AgNPs/TCNQ composites.

## 3. Results & Discussion

### 3.1. Thickness of PEBAX/AgNPs/TCNQ Composite Membranes

**Figure 1:**
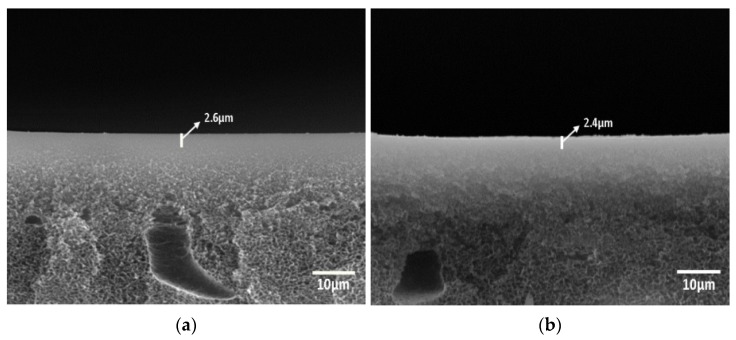
SEM images of (**a**) PEBAX-5513/AgNPs/TCNQ and (**b**) PEBAX-1657/AgNPs/TCNQ membranes.

The SEM images (Figure 1) of PEBAX-5513/AgNPs/TCNQ and PEBAX-1657/AgNPs/TCNQ membrane showed the thickness of the membrane and surface state. The SEM images indicated that the PEBAX/AgNPs/TCNQ composite solutions were coated on polymer support (polysulfone, sponge-like structure) without deep penetration. The selective layer of PEBAX/AgNPs/TCNQ composites was confirmed as (a) 2.6 µm and (b) 2.4 µm, respectively.

### 3.2. Separation Performance

**Figure 2:**
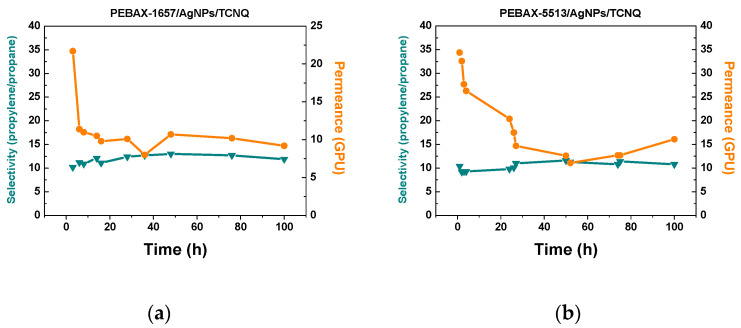
Long term gas separation performance of (**a**) PEBAX-1657/AgNPs/TCNQ and (**b**) PEBAX-5513/AgNPs/TCNQ.

The experiment of long-term mixed gas permeation was conducted to compare the effect of the functional group ratio of the PEBAX polymer as shown in Figure 2. Each of these experiments was performed more than three times. The different separation performance of PEBAX-1657/AgNPs/TCNQ and PEBAX-5513/AgNPs/TCNQ. The mixed-gas permeance of PEBAX-1657/AgNPs/TCNQ composite membranes was 9.2 GPU. However, for the case of PEBAX-5513/AgNPs/TCNQ composite membranes, the permeance for the propylene/propane mixture was 16.1 GPU. Unlike the expectation that the permeance of PEBAX-1657 containing a higher percentage of flexible PE groups would be higher, a higher permeance of PEBAX-5513 was observed. To confirm the reason why PEBAX-5513 showed the higher permeance, FT-IR and UV analysis were investigated.

### 3.3. Coordinative Interactions

**Figure 3:**
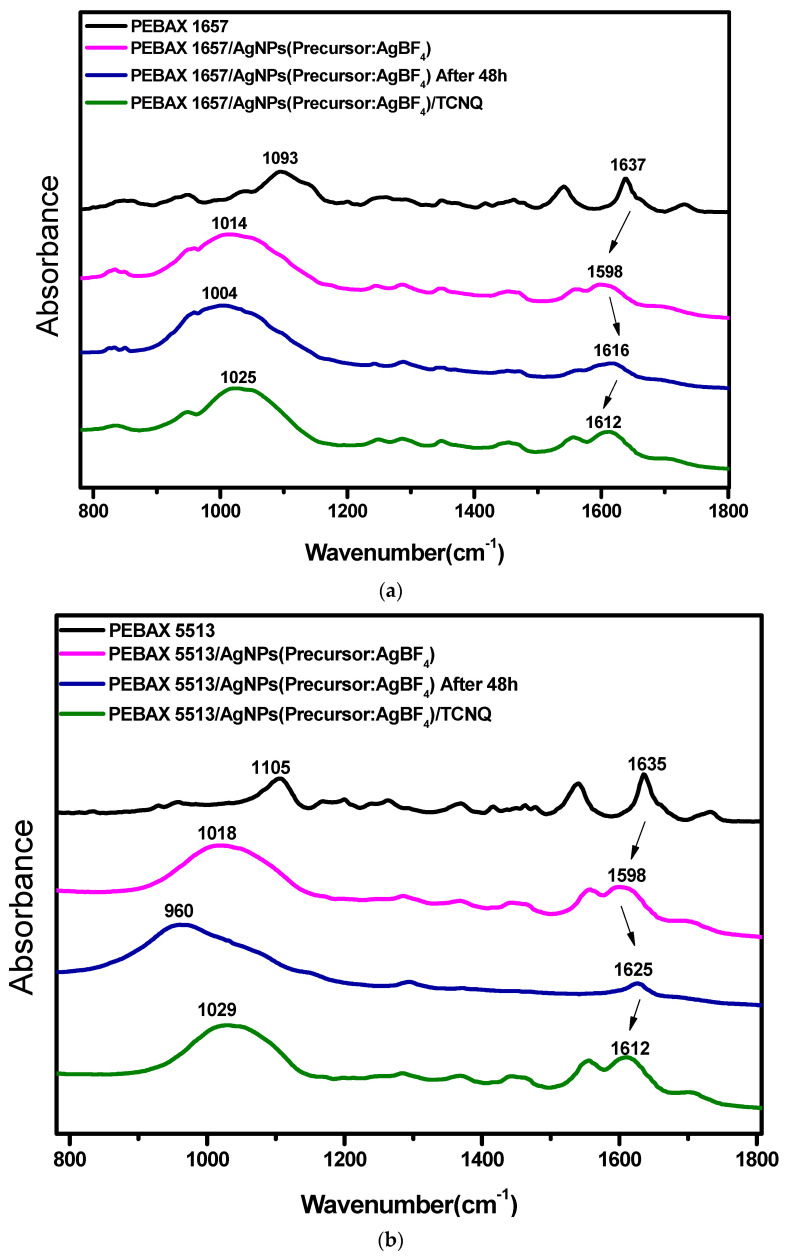
FT-IR spectra of (**a**) PEBAX-1657 and (**b**) PEBAX-5513 composites.

Figure 3 shows the interactions between silver ions/particles with PEBAX-1657 and PEBAX-5513, and it was analyzed through FT-IR spectroscopy. Free C=O (carbonyl bonds in the polyamide group) stretching bands of neat PEBAX-1657 were observed at 1637 cm^−1^. When AgNPs generated from AgBF_4_ were integrated into the membranes, the stretching bands of C=O shifted from 1637 to 1598 cm^−1^ by the remaining silver ions (not reduced). However, when the membrane was measured after 48 h, the stretching bands shifted from 1598–1616 cm^−1^ (PEBAX-1657) and 1625 cm^−1^ (PEBAX-5513). These results indicated that the silver ions became reduced over time and thus the interaction with carbonyl groups became weakened. Finally, when TCNQ was added, the bonds of the carbonyl groups became stronger due to the effect of positively polarizing the surface of AgNPs, and the peak shifted to 1612 cm^−1^.

For the case of the polyether group, when AgNPs generated from AgBF_4_ and TCNQ were integrated into the PEBAX-1657 membrane, the peak of the wavenumber shifted from 1093–1025 cm^−1^ (shift: 68 cm^−1^). However, when AgNPs generated from AgBF_4_ and TCNQ were integrated into the PEBAX-5513 membrane, the peak of the wavenumber shifted from 1105–1029 cm^−1^ (shift: 80 cm^−1^). It was a bigger shift in PEBAX-5513, unlike the expectation that the bigger shift in PEBAX-1657 would be observed due to large percentage of PE groups. The results suggested that the relatively small amount of PE groups in PEBAX-5513 were able to bind more strongly to AgNPs than those of PEBAX-1657 with a larger amount of PE group.

**Figure 4:**
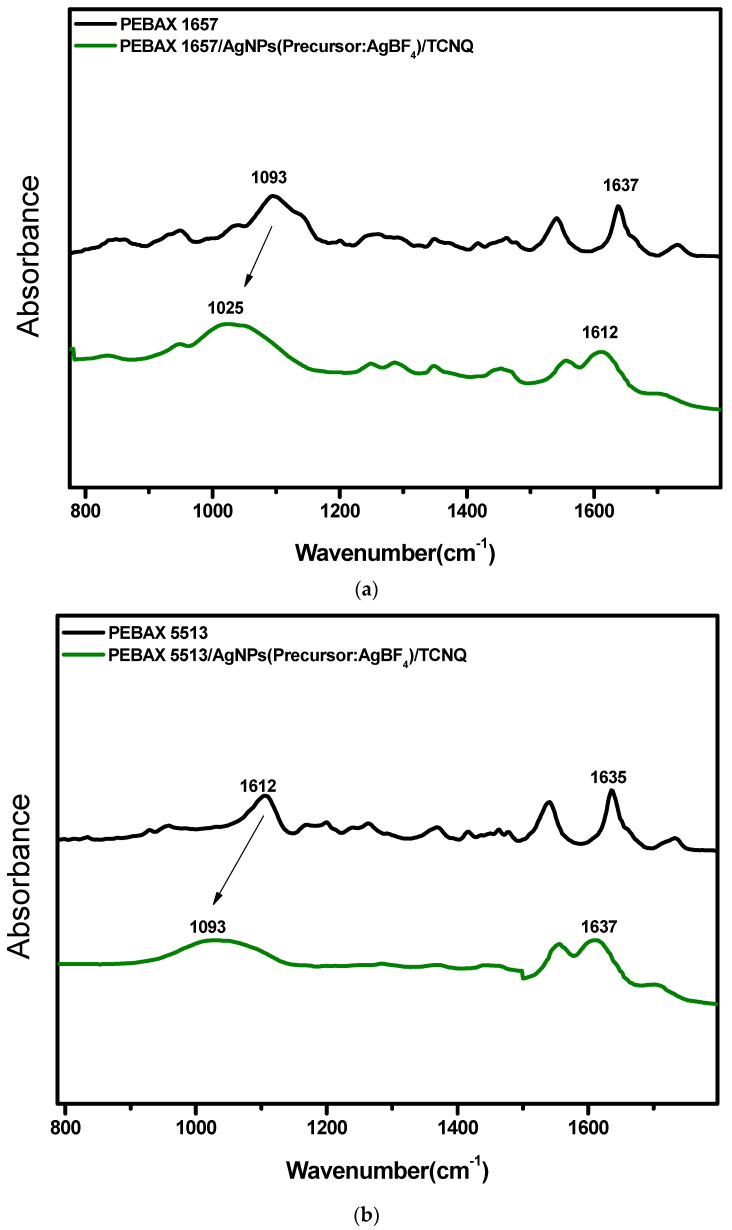
FT-IR spectra for polyether group of (**a**) PEBAX-1657 and PEBAX-1657/AgNPs, (**b**) PEBAX-5513 and PEBAX-5513/AgNPs.

**Figure 5:**
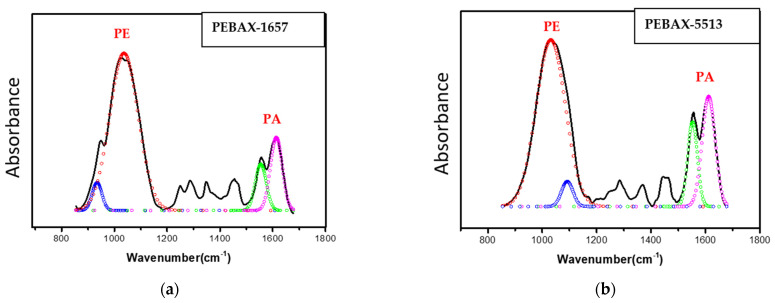
Deconvoluted FT-IR spectra of (**a**) PEBAX-1657 and (**b**) PEBAX-5513.

The FT-IR spectra of the membranes were deconvoluted to investigate the proportion of ether groups and carbonyl groups in both PEBAX-1657 and PEBAX-5513 as shown in Figure 4. Furthermore, Figure 5 and Table 1 showed that PEBAX-5513 has a relatively larger proportion of PA groups than PEBAX-1657. Thus, it could be thought that a higher proportion of PA group in PEBAX-5513 could more effectively stabilize AgNPs. From these results, we confirmed the effect of functional groups on stabilization and polarization on the surface of Ag nanoparticles, resulting in the enhancement of separation performance.

### 3.4. UV-Visible Absorption Spectra

**Figure 6:**
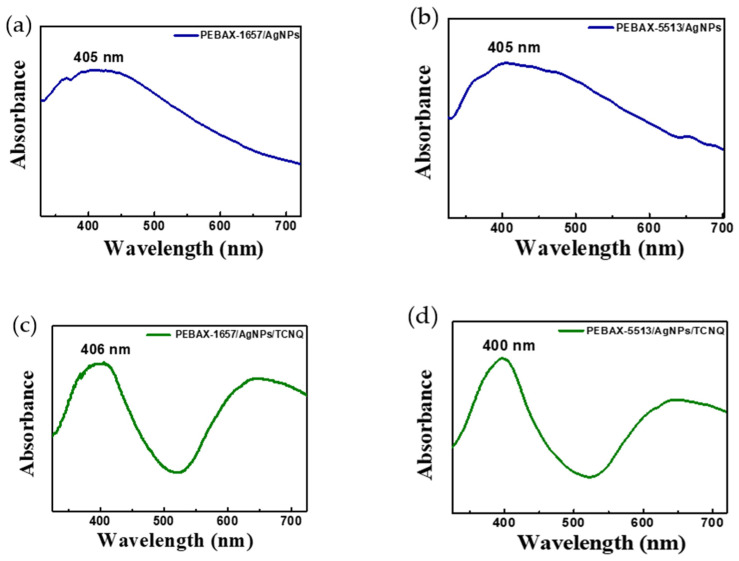
UV-visible absorption spectra of AgNPs in (**a**) PEBAX-1657/AgNPs, (**b**) PEBAX-5513/AgNPs, (**c**) PEBAX-1657/AgNPs/TCNQ, and (**d**) PEBAX-5513/AgNPs/TCNQ composites.

The size of the AgNPs was confirmed using UV-vis spectroscopy since it was known that the 420 nm indicated an absorption peak of AgNPs by plasmon excitation. Figure 6 shows that the highest peaks were observed at 400~406 nm, indicating the formation of Ag nanoparticles since NPs were more sensitive to UV-vis rays than organic molecules even though the UV-Vis spectra of TCNQ solution was observed at 400 nm, 300–370 nm, and 600–900 nm.

Figure 6a,b shows the asymmetrical peak in PEBAX-1657/AgNPs and PEBAX-5513/AgNPs, respectively, and the particle size was not uniform when TNCQ were not added in the composites. However, when TCNQ were added to PEBAX-1657/AgNPs or PEBAX-5513/AgNPs, the peak in the UV-vis spectrum became symmetrical, indicating that the particle size was uniform and stable in the composites by the stabilization effect of TCNQ on the AgNPs.

**Figure 7:**
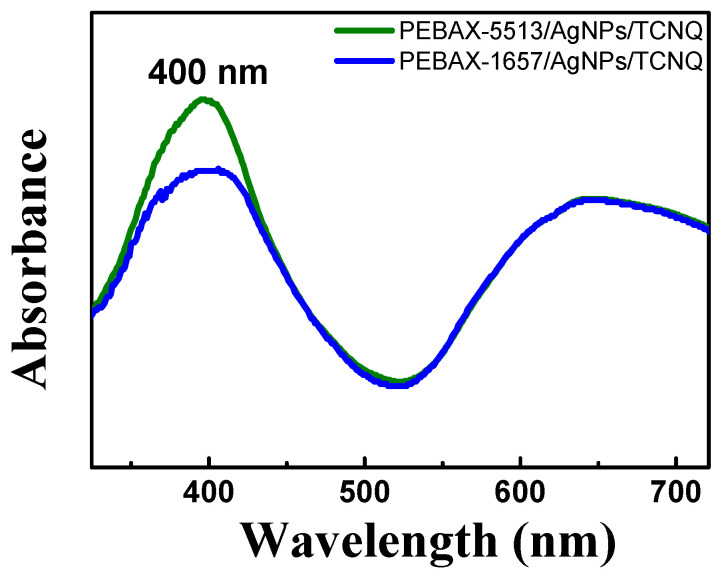
UV-visible absorption spectra of AgNPs in PEBAX-1657/AgNPs/TCNQ and PEBAX-5513/AgNPs/TCNQ composites.

**Scheme 1:**
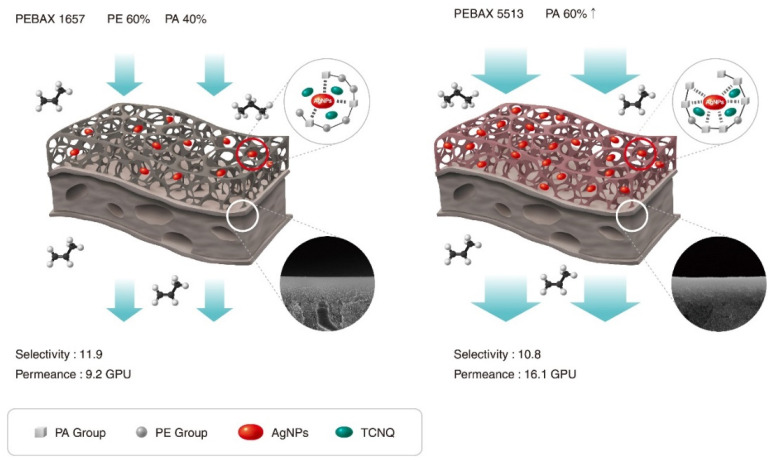
Formation of AgNPs in PEBAX-1657/AgNPs/TCNQ and PEBAX-5513/AgNPs/TCNQ composites.

To compare the formation of AgNPs in PEBAX-1657/AgNPs/TCNQ and PEBAX-5513/AgNPs/TCNQ, UV-vis spectra data was analyzed to confirm the quantity of AgNPs as shown in Figure 7. The absorption peak of PEBAX-5513/AgNPs/TCNQ was observed as having higher absorbance than that of PEBAX-1657/AgNPs/TCNQ, indicating that abundant PA groups of PEBAX-5513 could form the uniformly stable AgNPs capable of reversibly interacting with olefin molecules. As a result, the generated silver nanoparticles could play a role in improving the permeance of the membrane as shown in Scheme 1.

### 3.5. TGA Analysis

**Figure 8:**
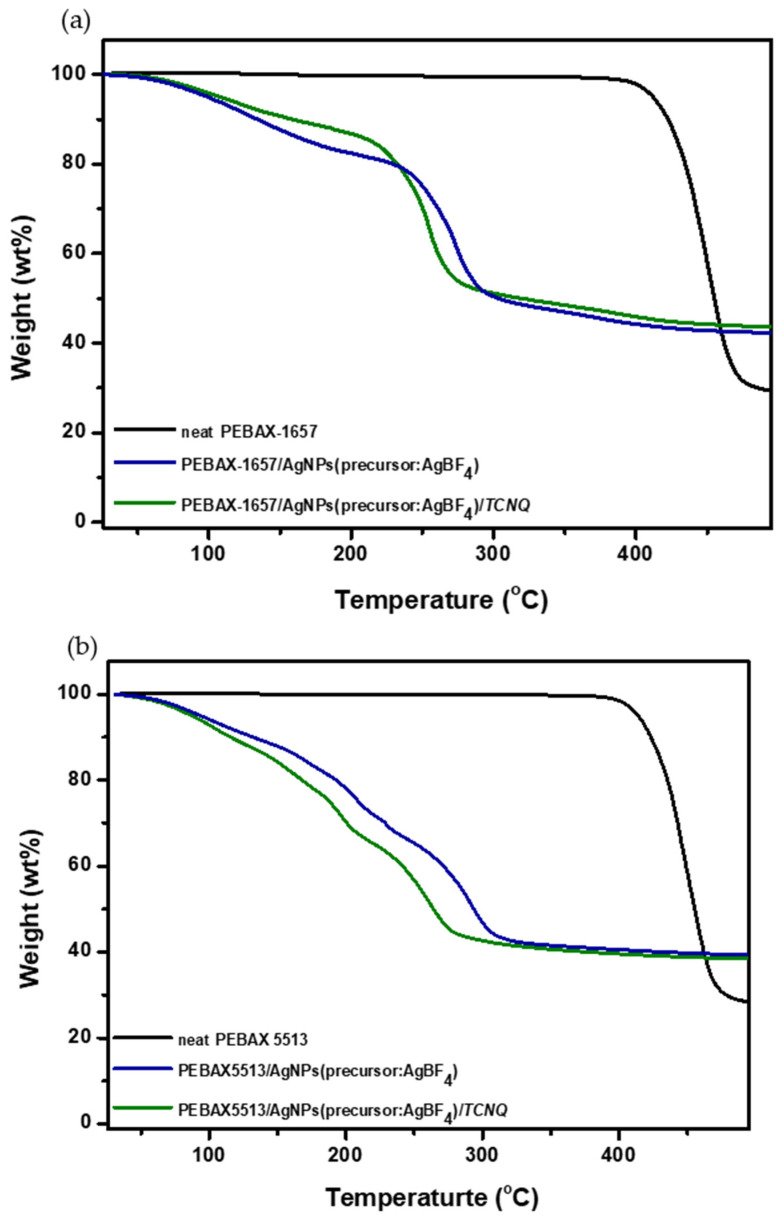
TGA thermograms of (**a**) PEBAX-1657, PEBAX-1657/AgNPs, and PEBAX-1657/AgNPs/TCNQ, and (**b**) PEBAX-5513, PEBAX-5513/AgNPs, and PEBAX-5513/AgNPs/TCNQ.

The thermal properties in both PEBAX-1657 and PEBAX-5513 composite membranes were analyzed by TGA in Figure 8. For the case of PEBAX-1657, when AgNPs were generated into the PEBAX-1657 polymer, the membrane with AgNPs showed the initial weight loss at 80–120 °C. The second weight loss occurred at 230–250 °C, degrading the PEBAX-1657 membrane. When TCNQ were added, the initial thermal stability was improved due to the TCNQ cross-linked with PEBAX-1657 chain. However, the second weight loss occurred at 210–230 °C, indicating that the thermal stability decreased and plasticization of the membrane occurred. This was attributable to positive polarized surface of AgNPs by electron acceptor TCNQ. When AgNPs were generated in the PEBAX-5513 polymer, unlike PEBAX-1657, the thermal stability steadily decreased. This was due to the reduced AgNPs by stabilization effect of PA groups in PEBAX-5513 as well as the plasticization of the membrane by positive polarization when TCNQ was added. From these results, it was expected to be applied to various fields such as battery separator and fluorescence as well as separation [22].

## 4. Conclusions

The facilitated olefin transport membranes consisting of PEBAX-1657/AgNPs/TCNQ and PEBAX-5513/AgNPs/TCNQ composites were prepared and measured for separation performance. The composites were characterized using SEM, FT-IR, UV-visible spectroscopy, and TGA. The selectivity and permeance performance for the PEBAX-1657/AgNPs/TCNQ composite membranes were 11.9 and 9.2 GPU, respectively, while those of the PEBAX-5513/AgNPs/TCNQ composite membranes were 10.8 and 16.1 GPU, respectively. These separation performances for both PEBAX-1657/AgNPs/TCNQ and PEBAX-5513/AgNPs/TCNQ composites were attributable to the stably reduced and positively polarized AgNPs by electron acceptor TCNQ. Especially, to confirm these different values of separation performance, we proved the functional groups-effect on separation performance using FT-IR. Furthermore, the effect of the functional groups on silver nanoparticles generated was confirmed through the UV-vis. As a result, unlike the expectation that the permeance of PEBAX-1657 having 60% of PE group would be higher, it was confirmed that the PA group of the PEBAX-5513 contributed to the formation of positively polarized abundant AgNPs, resulting in the high permeance. These results could be expected to suggest the new molecular structure, as polymer matrix for facilitated olefin transport membranes show both high selectivity and high permeance.

## Figures and Tables

**Table 1:** Area percentage of each FT-IR peak in PEBAX-1657 and PEBAX-5513 composites.

	Ether Groups (Area %)	Carbonyl Groups (Area %)
PEBAX-1657	77.7	22.3
PEBAX-5513	69.7	30.3
